# Risk of herpes zoster after exposure to varicella to explore the exogenous boosting hypothesis: self controlled case series study using UK electronic healthcare data

**DOI:** 10.1136/bmj.l6987

**Published:** 2020-01-22

**Authors:** Harriet Forbes, Ian Douglas, Adam Finn, Judith Breuer, Krishnan Bhaskaran, Liam Smeeth, Simon Packer, Sinéad M Langan, Kathryn E Mansfield, Robin Marlow, Heather Whitaker, Charlotte Warren-Gash

**Affiliations:** 1 Faculty of Epidemiology and Population Health, London School of Hygiene and Tropical Medicine, London, WC1E7HT, UK; 2 Bristol Children’s Vaccine Centre, Schools of Population Health Sciences and of Cellular and Molecular Medicine, University of Bristol, Bristol, UK; 3 UCL Division of Infection and Immunity Wing 1.3, London, UK; 4 Field Epidemiology Services, Public Health England Bristol, Bristol, UK; 5 Statistics, Modelling and Economics Department, Data and Analytical Sciences, National Infection Service, Public Health England Colindale, London, UK

## Abstract

**Objective:**

To assess the magnitude and duration of any hypothesised protective effect of household exposure to a child with varicella on the relative incidence of herpes zoster in adults.

**Design:**

Self controlled case series.

**Setting:**

UK general practices contributing to Clinical Practice Research Datalink.

**Participants:**

9604 adults (≥18 years) with a diagnosis of herpes zoster (in primary care or hospital records) between 1997 and 2018, who during their observation period lived with a child (<18 years) with a diagnosis of varicella.

**Main outcome measures:**

Relative incidence of herpes zoster in the 20 years after exposure to a child with varicella in the household compared with baseline time (all other time, excluding the 60 days before exposure).

**Results:**

6584 of the 9604 adults with herpes zoster (68.6%) were women. Median age of exposure to a child with varicella was 38.3 years (interquartile range 32.3-48.8 years) and median observation period was 14.7 (11.1-17.7) years. 4116 adults developed zoster in the baseline period, 433 in the 60 days before exposure and 5055 in the risk period. After adjustment for age, calendar time, and season, strong evidence suggested that in the two years after household exposure to a child with varicella, adults were 33% less likely to develop zoster (incidence ratio 0.67, 95% confidence interval 0.62 to 0.73) compared with baseline time. In the 10-20 years after exposure, adults were 27% less likely to develop herpes zoster (0.73, 0.62 to 0.87) compared with baseline time. A stronger boosting effect was observed among men than among women after exposure to varicella.

**Conclusions:**

The relative incidence of zoster was lower in the periods after exposure to a household contact with varicella, with modest but long lasting protective effects observed. This study suggests that exogenous boosting provides some protection from the risk of herpes zoster, but not complete immunity, as assumed by previous cost effectiveness estimates of varicella immunisation.

## Introduction

Primary infection with varicella zoster virus causes varicella (known commonly as chickenpox), typically in children. Herpes zoster (or shingles) arises from reactivation of latent varicella zoster virus following reduced cell mediated immunity, some years after the primary infection. Uncomplicated zoster is typically a mild, self limiting condition, but complications can occur,^1^ some of which, such as encephalitis, lead to severe illness, high healthcare costs, and mortality,^2^ whereas others, such as post-herpetic neuralgia and Ramsay Hunt syndrome, can seriously affect quality of life.^2^ ^3^ ^4^ In 1965, Hope-Simpson proposed that immunity to the varicella zoster virus is boosted through exposure to varicella contacts,^5^ later called the exogenous boosting hypothesis, as well as asymptomatic reactivation of varicella zoster virus, called endogenous boosting. Although a varicella vaccine is available, in many countries, including 17 European countries (including the United Kingdom), New Zealand, and China, it is not part of routine childhood vaccination programmes.^6^ This is partly because of concerns that its introduction would lead to a temporary increase in the number of cases of herpes zoster for 30-50 years, following removal of circulating varicella zoster virus.^7^

Although not universally accepted, epidemiological risk factor studies assessing the risk of zoster after exposure to varicella, and immunological studies assessing varicella zoster virus specific immunity after exposure to varicella, provide some credence to the exogenous boosting hypothesis.^8^ ^9^ ^10^ What is less well understood is the degree and duration of any protection conferred from re-exposure to varicella on future risk of zoster. Some cost effectiveness analyses informing varicella vaccination policy assume that exogenous boosting provides complete protection from zoster for up to 20 years, after which individuals revert to full susceptibility (the temporary immunity hypothesis).^11^ Others, however, incorporate different assumptions^12^—for example, that exposure to varicella confers partial protection that wanes but can accumulate on repeated exposure (the progressive immunity hypothesis).^13^ Lack of understanding about the role of exogenous boosting has meant public health decision making relies on mathematical models based on varied, and largely unsubstantiated, assumptions.^13^ ^14^

Model based projections predict a higher incidence of zoster after varicella vaccination, resulting from a reduction in exogenous boosting, with zoster incidences peaking around 30 years after implementation in individual based models^15^ and 20 years in older deterministic models. Real-world evidence to date, however, does not entirely support these predictions. In the United States—the only country with a universal two dose varicella vaccination programme (introduced in 2007) of sufficient length for potential changes in zoster incidence to be observed—evidence suggests zoster began increasing before the introduction of the programme and continued to increase at similar rates after implementation.^16^ ^17^ In Australia, where a state funded one dose varicella vaccination programme has been in place since 2006, no increases in hospital admissions for zoster have been observed.^18^ This might be explained by low varicella vaccine coverage or initial insufficient one dose vaccine schedules, or both, allowing ongoing transmission of the varicella zoster virus with some exogenous boosting. Changes in zoster incidence might also be explained by demographic changes, such as declining birth rates^19^ and the introduction of a vaccine against zoster. The lack of the predicted increase in zoster after routine varicella vaccination questions whether varicella zoster virus boosting from exposure to varicella contacts is as important as thought at a population level.^8^ ^17^

Given the sparse empirical evidence about the exogenous boosting hypothesis, we carried out a self controlled case series to estimate the relative risk of zoster over time after exposure to a household contact with varicella.

## Methods

### Data source

Data were obtained from the UK Clinical Practice Research Datalink (CPRD)^20^ linked to the hospital episode statistics, Office for National Statistics death registrations, and index of multiple deprivation data. CPRD contains anonymised primary care records from around 9% of the UK population, registered at more than 700 general practices.^21^ Continuous CPRD data are available for each patient, including diagnoses (recorded using Read codes), prescriptions (recorded using British National Formulary codes), and basic demographic data. Individuals registered in CPRD are representative of the UK population for age and sex.^20^ About 80% of CPRD practices in England are eligible for linkage with hospital episode statistics, Office for National Statistics, and index of multiple deprivation data; data are linked by the trusted third party NHS Digital using deterministic methods.^22^ To maximise study power, in our primary analysis we included patients with and without linked data. Hospital episode statistics data contains all NHS funded hospital admissions in England since 1997, including diagnoses (coded using international classification of diseases, 10th revision).^20^ Index of multiple deprivation data provide patient level data by mapping patients’ postcode to geographical areas with predefined deprivation scores; index of multiple deprivation combines several indicators, chosen to cover a range of economic, social, and housing issues, into a single deprivation score.^20^

### Study design

We carried out a self controlled case series analysis (an overview of the method is provided elsewhere^23^), which is a relatively novel epidemiological study design where individuals act as their own controls. Comparisons are made within individuals rather than between individuals as in a cohort or case-control study. Thus, only those who have experienced both the outcome and the exposure of interest are included.^24^ ^25^ Self controlled case series investigate the effect of a time varying exposure on an outcome by comparing the incidence of adverse events within periods of hypothesised excess risk from exposure with incidence during all other times. The temporal association between an exposure and an event is estimated using Poisson models to derive incidence rate ratios, comparing the rate of the outcome during an individual’s periods of exposure and non-exposure. Self controlled case series have been applied in various settings and are particularly useful when an appropriate comparison group of unexposed individuals is difficult to identify.^23^

### Selection of participants and observation period

The source population comprised all adults with at least one day of registration with a CPRD practice meeting CPRD quality control standards between 1 April 1997 (ie, date from which linked hospital episode statistics data were available) to 31 July 2018. From the source population we identified those with a first ever diagnosis of zoster who additionally had a child (<18 years) in the household (same family practice number) with a varicella record. A new family practice number variable is generated by the general practice software when patients register with a general practitioner or move address, assigning the same number to all those with the same address (CPRD Knowledge Centre, personal communication, 2017).

We began the observation period at the latest of; 12 months after the beginning of CPRD follow-up^26^ (to ensure zoster diagnoses represented new cases, with CPRD follow-up defined as the latest of patient registration date at the practice and date at which the practice data met CPRD quality control standards^20^), the date patients became 18 years of age, or the 1 April 1997 (study start date). The end of the observation period was defined as the earliest of date of death, date on which patients left the practice, day of the last data collection from the practice, or 31 July 2018 (study end date).

**Fig 1:**
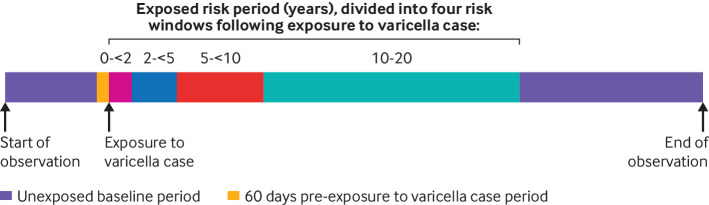
Graphical representation of framework for self controlled case series for an individual with more than 20 years of observation

Figure 1 illustrates the self controlled case series framework for an individual participant, including their pre-exposure period, risk periods, and unexposed observation periods.

*Pre-exposure period—*Patients with zoster are infectious during the blister phase, typically lasting 7-10 days from rash onset (with complete healing within 28 days),^27^ during which time they might infect a susceptible contact. The incubation period for varicella is 10-21 days.^28^ As such, any susceptible contact might develop varicella within 10 to 49 days of the zoster diagnosis. We therefore excluded a period of 60 days before exposure to varicella from baseline (unexposed) time, to allow for a zoster event resulting in an increased risk of varicella. The 60 day period was chosen to represent a conservative pre-exposure period (and variations were explored in post hoc sensitivity analyses).

*Risk (exposed) periods*—A priori we planned to have a fixed 20 year risk period, consistent with the duration of boosting assumed in the most conservative of cost effectiveness analyses of vaccines against varicella zoster virus.^11^ We subdivided exposure time into five risk windows (0-<2, 2-<5, 5-<10, 10-20 years) and used standard self controlled case series analytical techniques, where the incidence of zoster is assumed to be constant during a risk window, as risk of zoster might vary within this 20 year risk period.

*Baseline (unexposed) periods*—All other observation time made up the baseline (unexposed) period—that is, all time before 60 days before exposure to varicella in the household, and all time following the 20 years after exposure to varicella.

### Self controlled case series assumptions

The self controlled case series method relies on three key assumptions. Firstly, recurrent events (or outcomes) must be independent—that is, the chance of a second event is not influenced by having a first event. Zoster can recur (although recurrence is rare in people who are immunocompetent),^5^ ^29^ ^30^ ^31^ ^32^ and therefore this assumption could be violated; however, when the event is rare (which applies to zoster), considering only the first zoster episode is a valid approach to overcoming this potential bias.^24^ ^33^ Secondly, the occurrence of an event (zoster) should not alter the probability of subsequent exposure (to varicella). People with zoster are infectious during the blister phase and this might result in varicella if they are in direct contact with susceptible contacts. As zoster could lead to an increased incidence of varicella this might artificially inflate the relative rate of zoster events occurring in unexposed versus exposed (to varicella) periods. This can be overcome by removing a predefined period before (varicella) exposure from all other unexposed (baseline) time.^34^ Thirdly, the event of interest must not censor the observation period—for example, if the zoster event increases the likelihood of death. This assumption is fulfilled, as zoster is rarely associated with increased mortality.^35^ Supplementary box e-1 provides a detailed discussion of these fundamental assumptions.

### Exposures

*Household contact with varicella*—To identify child (<18 years) household contacts with varicella we searched for all varicella cases recorded in primary care (CPRD) and as part of a hospital admission (hospital episode statistics, any diagnostic field). We then used the family practice number to match these cases of varicella with cases of zoster. Exposure to varicella had to occur during the observation period of adults with zoster. If varicella was recorded in both hospital episode statistics and CPRD within the same contact, we used the earliest recorded diagnosis. For varicella cases identified in hospital episode statistics, we defined the date of varicella diagnosis as the date patients were admitted. To ensure we captured incident cases of varicella, we excluded those contacts without six months of CPRD follow-up, before their first varicella record.

*Household contacts with acute gastroenteritis*—We repeated the self controlled case series using household contacts with a child (<18 years) with acute gastroenteritis as a negative control exposure. Before the introduction of a routine vaccination programme in 2013, rotavirus was the most common cause of acute gastroenteritis. Rotavirus would not plausibly be expected to influence the future risk of zoster. Therefore, we did not expect to see a temporal association between exposure to gastroenteritis and zoster incidence; any association would call into question exogenous boosting as the interpretation of a positive result in the main analysis. We selected only gastroenteritis events up to 2012 (as rotavirus vaccination was introduced in 2013) and only the first episode of gastroenteritis. Acute gastroenteritis was defined using a comprehensive list of Read and hospital episode statistics codes, to identify primary care related (general practitioner diagnosed) and secondary care related (hospital diagnosed) events, respectively, until December 2012. Hospital episode statistics ICD-10 codes included A00-A09, for infectious intestinal diseases listed in any field. Read codes included infectious gastroenteritis and gastroenteritis of unspecified type (eg, combined diarrhoea and vomiting).^36^

### Outcome

The primary outcome was a first ever episode of acute zoster. We included zoster diagnosed in adults (≥18 years) during the observation period and excluded those with a history of post-herpetic neuralgia. We identified zoster in CPRD or hospital episode statistics (recorded in any diagnostic position of any episode of a hospital admission), using the earliest recorded diagnosis if zoster was recorded more than once. For zoster cases identified as part of a hospital admission, we defined the date of zoster diagnosis as the date the episode started (period of hospital admission under the care of a specific doctor).

### Covariates

A major strength of the self controlled case series design is that individuals serve as their own controls. The methodology therefore implicitly accounts for factors that remain constant over time (eg, sex). Consequently we captured only time varying covariates, age, calendar time, and season. We chose 40 age bands using quantiles (equal sized groups) of age at first zoster diagnosis.^37^ Calendar period was captured (using two year time periods) to adjust for changes in clinical, diagnostic, and administrative practices over the study period that could influence how exposures and outcomes were recorded. As varicella is more common in winter and spring, we captured season to account for any confounding effects, defined as winter (December-February), spring (March-May), summer (June-August), and autumn (September-November).

Age (<50 and ≥50 years, to crudely separate exposure of parent from that of grandparent), sex (men and women), and severe immunosuppression status at household exposure to varicella were assessed as effect modifiers. Severe immunosuppression was defined as conditions or drugs that are listed as contraindications for the live zoster vaccine in the UK, which was introduced in 2013 to limited age groups^38^; if vaccinated, those with primary or acquired immunodeficiency are considered to be at risk of developing a varicella-like or zoster illness from the live vaccine virus strain. Immunosuppressive diagnoses and drugs were identified in primary care (CPRD) and secondary care (hospital episode statistics, any diagnostic position) records and were time updated (that is, patient’s immunosuppression status could vary over his or her observation period: see supplementary box e-2 for more details). We also used information on quintiles of the patient level index of multiple deprivation to describe the cohort.

The morbidity codes used in this study are available for download: https://doi.org/10.17037/DATA.00001158.

### Statistical analyses

Data management and analyses were carried out in STATA/SE 15.1 and RStudio (self controlled case series package).^37^ We used conditional Poisson regression to calculate incidence rate ratios comparing the incidence of zoster after exposure to varicella with baseline (unexposed) time.^24^ We first adjusted for age (defined by 40 quantiles of age at zoster diagnosis), then added calendar time and season. If participants had multiple exposures to household contacts with varicella during their observation period, we considered only the first exposure in the primary analysis (multiple exposures were explored in a secondary analysis). We similarly ignored secondary episodes of zoster.

Secondary analyses included investigating whether the association between exposure to varicella and risk of zoster varied by age, sex, and severe immunosuppression status of the zoster case; investigating whether there was a dose-response effect with repeated varicella exposures (accounting for repeat exposures by ending the last day of a risk period the day before a subsequent exposure to varicella, see supplementary figure e-4 for graphical representation); and repeating the main self controlled case series analysis using gastroenteritis exposure as a negative control exposure. Finally, as the analyses suggested boosting might last beyond 20 years we chose not to explore shorter risk periods (as per protocol) but instead explored an indefinite risk period after exposure to varicella contacts.

### Sensitivity analyses

We checked our assumptions through several sensitivity analyses: 1) using a spline based age effect, where the relative age effect is represented by a smooth function obtained by splicing together polynomials of low dimension, to ensure we had fully adjusted for age effects; 2) restricting to patients with linked hospital episode statistics records only (as linked patients had better capture of our exposure and outcome); 3) restricting to households with a single child aged <16 years (to reduce the likelihood of misclassifying exposure time); 4) excluding those with recurrent zoster during the observation period (with the recurrent episode defined as a zoster record five years after first zoster record); 5) excluding those with a zoster vaccination; and 6) assessing hip fracture as an alternative outcome, which is strongly associated with increasing age (if the age adjustment in our primary analysis was sufficient, we should observe a null association between exposure to varicella and hip fracture). In post hoc analyses we varied the pre-exposure window, defining it as 30 and 90 days, to explore whether these different durations affected the study findings.

### Patient and public involvement

Neither patients nor the public were involved in developing the research question and study or in the design, management, or interpretation of this study.

## Results

Overall, 9604 adults with zoster were exposed to a child contact with varicella in the household during the study period (supplementary fig e-1), of whom 39 (0.4%) received a diagnosis of zoster in hospital. The median age at first zoster diagnosis was 41.1 years (interquartile range 33.1-51.3 years) and at first known exposure to varicella was 38.3 (32.3-48.8) years (table 1). The median age of the children at varicella episode was 3.8 (2.3-5.7) years. The median observation period was 14.7 (11.1-17.7) years. In total, 118 (1.2%) participants were severely immunosuppressed at the time of exposure to varicella. Participants’ ages varied widely at the start of the observation period (supplementary fig e-2) and at exposure to varicella and diagnosis of zoster (supplementary fig e-3).

**Table 1:** Characteristics of 9604 adults with zoster exposed to a child with varicella in the household. Values are numbers (percentages) unless stated otherwise

Characteristics	Estimates
Median (interquartile range) age at first zoster diagnosis (years)	41.1 (33.1-51.3)
Median (interquartile range) age of household varicella contact at diagnosis (years)	3.8 (2.3-5.7)
Median (interquartile range) age of exposure to household varicella contact (years)	38.3 (32.3-48.8)
Median (interquartile range) years of observation	14.7 (11.1-17.7)
Women	6584 (68.6)
Eligible for hospital episode statistics linkage	5481 (57.1)
Fifth of patient level index of multiple deprivation:	
1st (least deprived)	1345 (14.0)
2nd	1198 (12.5)
3rd	1124 (11.7)
4th	993 (10.3)
5th (most deprived)	876 (9.1)
Missing	4068 (42.4)
Start of observation period in adults:	
1997-99	3875 (40.3)
2000-04	3936 (41.0)
2005-09	1355 (14.1)
2010-15	407 (4.2)
2015-18	31 (0.3)
Season of exposure to household varicella contact:	
Winter (December-February)	2341 (24.4)
Spring (March-May)	3438 (35.8)
Summer (June-August)	2488 (25.9)
Autumn (September-November)	1337 (13.9)
Severely immunosuppressed at household exposure to varicella contact	118 (1.2)

In the unadjusted analysis, the relative risk of zoster was observed to increase 10-20 years after exposure, which would be expected, because as the risk period progresses people age and become more susceptible to zoster (table 2). After adjustment for the confounding effects of age, exposure to a household contact with varicella was associated with a reduced risk of zoster among adults for up to 20 years (table 2); adults were 33% less likely to develop zoster within two years of exposure to varicella (incidence ratio 0.67, 95% confidence interval 0.62 to 0.73) than during baseline (unexposed) time. The incidence ratio moved closer to 1 with time since varicella exposure; after exclusion of the baseline and pre-exposure groups, some evidence suggested a linear trend (P=0.03, comparison between a model with no exposure group and one with linear exposure). Ten to 20 years after exposure to a varicella contact, adults were 27% less likely to develop zoster (incidence ratio 0.73, 95% confidence interval 0.62 to 0.87) than during baseline (unexposed) time.

**Table 2:** Adjusted incidence ratios for zoster in 9604 adults during risk periods after household exposure to a child with varicella

Time period	No of zoster events	Person years of observation	Crude incidence ratio (95% CI)	Age adjusted incidence ratio* (95% CI)	Age, calendar time, and season adjusted incidence ratio* (95% CI)
Baseline†	4116	56 417	1.00	1.00	1.00
60 days pre-exposure	433	1539	3.17 (2.86 to 3.51)	2.89 (2.60 to 3.21)	2.87 (2.58 to 3.19)
Post-exposure risk period (years):					
0-<2	1177	18 031	0.77 (0.72 to 0.82)	0.68 (0.63 to 0.74)	0.67 (0.62 to 0.73)
2-<5	1432	22 2901	0.85 (0.80 to 0.91)	0.70 (0.64 to 0.77)	0.69 (0.63 to 0.76)
5-<10	1546	24 620	0.96 (0.89 to 1.02)	0.70 (0.63 to 0.79)	0.69 (0.61 to 0.77)
10-20	900	13 317	1.25 (1.14 to 1.37)	0.77 (0.65 to 0.90)	0.73 (0.62 to 0.87)

* Age defined by 40 quantiles of age at event (herpes zoster); calendar time defined as 1997-98, 1999-2000, 2001-02, 2003-04, 2005-06, 2007-08, 2009-10, 2011-12, 2013-14, 2015-16, 2017-18; and season defined as winter (December-February), spring (March-May), summer (June-August), and autumn (September-November).

† All time from observation start to 60 days pre-exposure, and after the 20 years after exposure: seven zoster events occurred after the 20 years after exposure.

The effect of exposure to varicella on risk of zoster varied between men and women (P<0.001 for interaction); varicella exposure was more protective among men than among women (table 3). We found no evidence of effect modification by immunosuppression status (P=0.53) or age at exposure to varicella (P=0.65).

**Table 3:** Incidence ratios for zoster in 9604 adults during risk periods after household exposure to a child with varicella, by other factors

Time period	No. of zoster events	Age, calendar time, and season adjusted incidence ratio* (95% CI)	P value for interaction†
**Sex**			
Men:			
Baseline‡	1354	1.00	P<0.001
60 days pre-exposure	188	3.58 (3.05 to 4.20)	
Years post-exposure:			
0-<2	336	0.55 (0.48 to 0.62)	
2-<5	417	0.57 (0.50 to 0.65)	
5-<10	464	0.57 (0.49 to 0.67)	
10-20	261	0.59 (0.48 to 0.74)	
Women:			
Baseline‡	2762	1.00	
60 days pre-exposure	245	2.49 (2.17 to 2.85)	
Years post-exposure:			
0-<2	841	0.74 (0.68 to 0.81)	
2-<5	1015	0.76 (0.69 to 0.84)	
5-<10	1082	0.76 (0.67 to 0.86)	
10-20	639	0.82 (0.68 to 0.98)	
**Age at household exposure to varicella (years)**			
18-49:			
Baseline‡	2834	1.00	P=0.65
60 days pre-exposure	350	2.90 (2.58 to 3.26)	
Years post-exposure:			
0-<2	924	0.66 (0.61 to 0.72)	
2-<5	1133	0.68 (0.62 to 0.75)	
5-<10	1288	0.69 (0.60 to 0.78)	
10-20	808	0.75 (0.63 to 0.90)	
≥50:			
Baseline‡	1282	1.00	
60 days pre-exposure	83	2.72 (2.16 to 3.42)	
Years post-exposure:			
0-<2	253	0.72 (0.61 to 0.83)	
2-<5	299	0.74 (0.63 to 0.87)	
5-<10	258	0.70 (0.56 to 0.86)	
10-20	92	0.67 (0.48 to 0.93)	
**Immunosuppression status at exposure to varicella **			
Immunocompetent:			
Baseline‡	4056	1.00	P=0.53
60 days pre-exposure	424	2.84 (2.55 to 3.16)	
Years post-exposure:			
0-<2	1162	0.67 (0.62 to 0.73)	
2-<5	1413	0.69 (0.63 to 0.75)	
5-<10	1537	0.69 (0.61 to 0.77)	
10-20	894	0.74 (0.62 to 0.87)	
Immune suppressed:			
Baseline‡	60	1.00	
60 days pre-exposure	9	4.74 (2.30 to 9.79)	
Years post-exposure:			
0-<2	15	0.74 (0.41 to 1.35)	
2-<5	19	0.95 (0.53 to 1.71)	
5-<10	9	0.52 (0.23 to 1.18)	
10-20	6	0.94 (0.33 to 2.68)	

* Age defined by 40 quantiles of age at event (herpes zoster); calendar time defined as 1997-98, 1999-2000, 2001-02, 2003-04, 2005-06, 2007-08, 2009-10, 2011-12, 2013-14, 2015-16, 2017-18; and season defined as winter (December-February), spring (March-May), summer (June-August), and autumn (September-November).

† Calculated using likelihood ratio test.

‡ All time from observation start to 60 days pre-exposure, and after the 20 years after exposure.

In total, 7972 (83.0%) participants had one recorded exposure to a household contact with varicella during the observation period, 1449 (15.1%) had two exposures, and the remaining 183 (1.9%) had three or more exposures. The median time between first and second exposure was 127 days (interquartile range 14-1541 days). Repeated household exposure to varicella was associated with a reduced risk of zoster, but no more than the reduced risk observed for the first exposure (table 4).

**Table 4:** Adjusted incidence ratios for zoster in 9604 adults during risk periods for those with one, two, or three or more recorded household varicella exposures

Varicella exposure and time period	No. of zoster events	Age, calendar time, and season adjusted incidence ratio* (95% CI)
Baseline†	4114	1.00
**1st exposure**		
60 days pre-exposure	429	2.90 (2.61 to 3.23)
Years post-exposure:		
0-<2	1149	0.67 (0.62 to 0.72)
2-<5	1372	0.70 (0.64 to 0.76)
5-<10	1431	0.69 (0.62 to 0.77)
10-20	811	0.75 (0.64 to 0.87)
**2nd exposure**		
60 days pre-exposure	24	1.64 (1.09 to 2.48)
Years post-exposure:		
0-<2	53	0.60 (0.44 to 0.81)
2-<5	63	0.73 (0.55 to 0.98)
5-<10	50	0.68 (0.48 to 0.96)
10-20	87	0.80 (0.59 to 1.07)
**≥3rd exposure**		
60 days pre-exposure	<5‡	0.59 (0.08 to 4.23)
Years post-exposure:		
0-<2	<5‡	0.34 (0.10 to 1.10)
2-<5	<5‡	0.39 (0.12 to 1.29)
5-<10	7	1.16 (0.47 to 2.86)
10-20	7	1.42 (0.52 to 3.85)

* Age defined by 40 quantiles of age at event (herpes zoster); calendar time defined as 1997-98, 1999-2000, 2001-02, 2003-04, 2005-06, 2007-08, 2009-10, 2011-12, 2013-14, 2015-16, 2017-18; and season defined as winter (December-February), spring (March-May), summer (June-August), and autumn (September-November).

† All time from observation start to 60 days pre-exposure, and after the 20 years after exposure.

‡ Cell counts less than five have been suppressed to preserve anonymity.

Exposure to a household contact with gastroenteritis was not associated with zoster (supplementary tables e-1 and e-2); within two years of exposure to gastroenteritis, no evidence was found of a decreased risk of zoster (incidence ratio 0.95, 95% confidence interval 0.87 to 1.04). When the risk period was defined as indefinite after exposure to varicella (that is, all observation time after exposure to varicella, not limited to 20 years after exposure to varicella), no evidence of an association was found between exposure to varicella and risk of zoster 20 years after exposure (incidence ratio 1.21, 95% confidence interval 0.54 to 2.74; see supplementary table e-3); however, the confidence intervals were wide, as only seven zoster events occurred.

### Sensitivity analyses

When modelling age as a spline based function, the effect of exposure to varicella on risk of zoster was consistent with the main analysis, suggesting adequate adjustment for age (supplementary table e-4). Excluding those without linked hospital episode statistics data (n=4123), those with recurrent zoster (n=132), and those receiving the live zoster vaccine (n=212) made little difference to the results (supplementary table e-4). In single child households, the point estimates were all closer to 1 (supplementary table e-4). Although exposure to varicella appeared to be associated with increased risk of hip fracture, after adjustment for age in 40 quantiles, the association was much attenuated (supplementary tables e-5 and e-6). Finally, shortening the pre-exposure window to 30 days strengthened the relative risk of zoster, whereas lengthening the window to 90 days attenuated the relative risk. When the pre-exposure time window was varied, however, the relative risks for the individual risk windows were broadly similar to those of the main analyses (supplementary table e-7).

## Discussion

This self controlled case series study found a reduced risk of zoster associated with exposure to a household contact with varicella, with modest but long lasting protective effects observed. Strong evidence suggests that in adults the risk of zoster within two years of exposure to a child with varicella in the household was 33% lower than in the baseline period, and in the 10-20 years post-exposure the risk was 27% lower. No evidence was found to indicate that protection against reactivation of the varicella zoster virus accumulates on repeated exposure (the progressive immunity hypothesis); however, the study might have been underpowered to detect an effect. These data challenge the assumption previously used in public health policy modelling in certain countries, including the UK, that through exogenous boosting people exposed to varicella are completely immune to zoster for between two and 20 years.^39^

### Strengths and limitations of this study

In this study we used a self controlled case series design to test the duration of varicella zoster virus exogenous boosting, which enabled us to control for confounding between participants and capture the precise timing of the included varicella and zoster diagnoses. Although our research question potentially violates some of the assumptions of self controlled case series methodology, we used recommended approaches to tackle these issues, including studying only first events and including a pre-exposure period in the analyses (supplementary box e-1). We controlled for the confounding effects of age, as evidenced by our negative outcome analysis (exploring the link between varicella contact and hip fracture); after exposure to varicella the relative rate of hip fractures was observed to increase substantially over time, but after adjustment for age as a spline based effect, the associations were no longer evident.

Capture of zoster cases is likely to be reliable and comprehensive. Zoster is typically a straightforward diagnosis, as it has a characteristic dermatomal distribution. Evidence suggests that most people with zoster seek out healthcare: a US based health and retirement survey among adults aged 55 and older reported that more than 91% who self reported herpes zoster had sought medical care^40^; another US based survey about immunisation practices in the US among adults aged 60 and older found that 95% of those who knew they had zoster sought care^41^; and a community based retrospective survey in Beijing, China (all ages) found that 92.4% of participants had sought healthcare for zoster.^42^ Varicella is similarly a straightforward diagnosis, so our records are likely to be reliable. We used a population based sample, representative of the UK population for age, sex, and ethnicity^20^ and therefore our findings are likely to be generalisable to the UK population.

This study also has some limitations. Varicella is likely to be under-ascertained in UK electronic health records; varicella tends to be a mild, self limiting condition that does not require a visit to the doctor, and consultations with doctors for varicella have been declining in the UK (between 2004 and 2014 by around 20% in 1-3 year olds and 6% in 4-6 year olds).^43^ This misclassification of varicella exposure might result in some true exposed time being misclassified as unexposed time (if the household contacts with varicella did not attend their general practice or our observation period did not cover the time of first exposure) and could therefore bias the effect estimate towards the null, thus potentially underestimating the effect of exogenous boosting. We were not able to capture occupational exposure to varicella, such as in healthcare or childcare workers: about two million people, or 5% of the population of England, during their work could be exposed to children with varicella.^44^ ^45^ Household cases of varicella are, however, likely to cluster together; studies suggest that about 78% of healthy household contacts develop varicella.^46^ In this case, if only a second or subsequent child presented to the doctor, the true first exposure would likely be in the 60 day pre-exposure period (because household clusters tend to occur within a few weeks of each other). This would mean that our current estimate for 0-<2 years is biased away from the null—that is, we have overestimated boosting effects in the 0-<2 years’ risk period.

Owing to the under-ascertainment of varicella in medical records, it is possible that those represented in general practice records might have more severe varicella. If this was the case, we would expect the cases to have a relatively higher viral load than the average individual with varicella, and potentially this would result in a greater degree of boosting in exposed adults. Therefore, by capturing more severe varicella cases, our study might have overestimated the degree of exogenous boosting.

We did not validate the family number variable (which identifies all those living in the same household), so we might have wrongly identified those as living in the same household. People can move in or out of households and people living together could be registered at different practices. This misclassification of varicella exposure is likely to be non-differential for zoster and thus would bias the effect estimates towards the null. However, we ensured that exposure to varicella occurred during the observation period of the participant with zoster to reduce the misclassification of varicella. We also removed diagnoses of varicella recorded in the six months following a person’s registration at their general practice, to ensure varicella diagnoses were incident and that we were not capturing historical varicella recorded as part of practice registration.

CPRD registered patients leaving one CPRD practice and entering another are not currently identifiable in the database. In theory therefore it is possible for patients to be entered twice in CPRD and thus appearing as two different people. However, patients’ medical records are transferred to their new practice. Therefore, any such patient would have a record, and therefore a history, of zoster before CPRD follow-up and be excluded from our study population during the time they were in their second practice. Furthermore, the rate of recurrent zoster is low (estimated to be between 1% and 6%)^5^ ^29^ ^30^ ^31^ ^32^; therefore if patients have a diagnosis of zoster recorded within one medical practice and transfer to another in CPRD, they are unlikely to have been included in our study again as their risk of varicella zoster virus being reactivated again is low.

We found no evidence of effect modification by immunosuppression status. It could be hypothesised that patients with severe immunosuppression might not experience the same degree of exogenous boosting, compared with immunocompetent patients, when exposed to a child with varicella; if their immune system is weakened, they may be unable to mount an effective immune response. Unfortunately, our study was underpowered to detect a difference in the boosting effect among immunosuppressed people, as only 1.2% of our study sample were immunosuppressed at exposure to varicella.

The study design required patients with zoster to be living with a child with varicella, therefore the study cohort is younger than a general population with zoster. If younger adults are more likely to attend their general practice only when zoster is severe, this could affect the generalisability of our study by making it more applicable to how exposure to varicella in the household protects against severe cases of zoster. However, when we restricted our analysis to adults aged 50 and older at exposure to varicella, a similar pattern of association was observed, with no evidence of effect modification by age (table 3). This suggests that although the median age of our study cohort (at exposure to varicella) was low, the findings can be generalised to older people.

Finally, bias could be introduced if inclusion in our study (that is, visits to a general practitioner for zoster) is differential according to the timing of exposure to a household contact with varicella. All our comparisons were within person, therefore health seeking biases are less likely. However, an individual’s health seeking behaviour might vary over time. It is plausible that parents caring for children with varicella might be less likely to attend a general practice during the acute phase of zoster owing to child care responsibilities. This would result in slightly lower ascertainment of zoster in the week after a diagnosis of varicella, which could have pulled the association between exposure to varicella and risk of zoster towards the null for the first risk period (0-<2 years post-exposure).

### Comparison with other studies

In a systematic review of epidemiological studies investigating the reduction in risk of zoster from exposure to varicella, 27 of 40 studies showed evidence of varicella zoster virus exogenous boosting, although the duration and magnitude of boosting could not be determined.^8^ The review identified nine epidemiological risk factor studies that investigated the risk of zoster after exposure to children generally and to children with varicella, four of which found evidence of varicella zoster virus exogenous boosting .^8^ Studies found that the degree of protection from exposure to children ranged from a 25%^11^ to 70%^47^ reduced risk, with studies using different time frames and numbers of exposures to varicella. In our study we used medical record data on the timing of exposure to both zoster and varicella, and thus might offer more reliable estimates of effect than studies relying on patient recall^47^ or using living with children as a proxy for exposure to varicella contacts.^11^ The review also included four prospective immunological studies analysing immunity to varicella zoster virus after exposure to varicella, measured using antibody titres; however, only two studies had data for up to one year after exposure. One longitudinal study showed that among grandparents re-exposed to varicella, the associated increased immune response to varicella zoster virus (measured as varicella zoster virus specific T cell immunity and antibody titres) was not universal and was noticeable for less than one year.^9^ Our results, indicating that exposure to a varicella contact might protect against zoster for over two years, may reflect the difficulty in measuring the immune response to varicella zoster virus.

To date no clear-cut increase in zoster incidence attributed to routinely vaccinating against varicella has been observed, as predicted by modelling studies incorporating the exogenous boosting hypothesis.^17^ The US has had a two dose varicella vaccination programme since 2007 and therefore could serve as a comparison to modelling predictions; here zoster incidence began increasing before the introduction of the vaccine.^16^ However, estimates of the timing of the first measurable increase in zoster vary according to the type of model and its assumptions, thus although the US has only seen a similar increase in zoster incidence as before varicella vaccination, it is possible that the “real” effect might only occur after more than 20 years from the vaccination programme being introduced. If, however, boosting does not provide complete immunity, as suggested by this self controlled case series, then the population impact of varicella vaccination on zoster incidence might not be as great as some model based studies predicted.^15^ A recent systematic review and meta-analysis of varicella vaccination and risk of zoster concluded that although exogenous boosting is plausible at an individual level, no conclusive evidence exists to date that varicella vaccination has a substantial population level impact on zoster.^17^

A priori, we hypothesised that as women are more likely to be the primary caregiver and thus have more direct physical contact with children with varicella, the degree of protection gained from boosting would be greater among women. We found the opposite. This could suggest that men mount a more effective immune response when boosted by exposure to a varicella case; most childhood infections are more severe in boys than in girls,^48^ therefore this could lead to greater immune memory and greater potential for boosting. It is also well established that zoster occurs more often in women,^49^ supporting the hypothesis of lower residual immunity in women. Alternatively, women may have more exposures to children outside the household, so the household boosting effect in men is therefore more pronounced.

### Conclusions and policy implications

These findings are important and timely given that certain countries (such as the UK^12^) are currently reviewing their varicella and zoster vaccination policy; this study suggests that in adults varicella zoster virus exogenous boosting does not provide complete immunity and therefore cost effectiveness analyses modelling the effect of varicella vaccination on zoster need to be revisited.

Future studies should substantiate these findings using different data sources; particular design improvements could be larger samples, to capture more individuals with repeated exposures to varicella (and thus better understand whether exogenous boosting with varicella zoster virus provides a fixed level of immunity or progressive immunity against zoster) and existing immunosuppression, older age samples with more adults aged 50 years and older where the burden of zoster exists, as well as data with better capture of exposure to varicella outside the household, particularly among grandparents.

This self controlled case series investigating the protective effect of exposure to a varicella case suggests exposure is associated with a 33% reduction in zoster incidence in the first two years, with some indication this effect wanes slightly but is maintained over the 20 years from exposure to varicella. These findings are themselves unable to justify for or against specific vaccination schedules, but they do suggest that revised mathematical models are required to estimate the impact of varicella vaccination, with the updated assumption that exogenous boosting is incomplete and only reduces the risk of zoster by about 30%.

**What is already known on this topic**

Hope-Simpson’s exogenous boosting hypothesis (that exposure to children with varicella during adulthood boosts immunity to varicella zoster virus, thereby preventing zoster) has gained widespread credence from epidemiological studies, but more recent immunological data suggest that boosting may not be long lasting
Epidemiological data to support the findings from immunological studies are limited
The hypothesis is important for policymakers relying on cost effectiveness analyses to make recommendations about introducing universal childhood varicella vaccination so is particularly relevant to the UK

**What this study adds**

In this self controlled case series study of UK adults with both household exposure to varicella and an episode of zoster, strong evidence suggests that exposure to varicella is associated with a reduction in risk of zoster by around 30% over 20 years
These findings cannot be used to justify for or against specific vaccination schedules
They do, however, suggest that previous mathematical models, estimating the effect of exogenous boosting in childhood varicella vaccination policy in the UK, that assume complete immunity for between two and 20 years may need revisiting

## Web extra

Extra material supplied by authors

## Notes

Ethical approval: This study was approved by the independent scientific advisory committee (19_026).

Data sharing: No additional data available.

The lead author (HF) affirms that this manuscript is an honest, accurate, and transparent account of the study being reported; that no important aspects of the study have been omitted; and that any discrepancies from the study as planned (and, if relevant, registered) have been explained.

Dissemination to participants and related patient and public communities: We will be sharing our results with the general public through a press release issued by the London School of Hygiene and Tropical Medicine and taking part in media interviews where possible. We will also write a blog post on the Electronic Health Records research group website at the London School of Hygiene and Tropical Medicine, which will be tagged on the research groups Twitter feed.
