# Supplementary material for: Risk of herpes zoster after exposure to varicella to explore the exogenous boosting hypothesis: self controlled case series study using UK electronic healthcare data
Source: BMJ. 2020 Jan 22;368:l6987. doi: 10.1136/bmj.l6987 (PMC7190015; doi:10.1136/bmj.l6987)
Supplement: Supplementary file 1 — Supplementary material: Additional information [file forh051402.ww1.pdf]

Supplementary material

Box e-1: SCCS assumptions

The SCCS method relies on three key assumptions.

1. **Recurrent events (i.e outcomes) must be independent**—that is, the chance of a second event is not influenced by having a first event. Zoster is occasionally recurrent; therefore, this assumption may be violated. Quantifying the proportion of zoster patients with recurrent zoster is challenging, as there is no formal definition of the time gap between the first and second zoster event, which signifies two separate events. However, estimates of recurrences range from 1-6%[1-5] and are more likely in immunocompromised patients.[1] Indeed in our study, only 132 (1.4%) of the 9604 included in the cohort had a second episode of zoster during their observation period. A valid approach to overcome this potential bias is to consider only the first incident zoster episode, if the event itself is rare.[6 7]
2. **The occurrence of an event (zoster) should not alter the probability of subsequent exposure.** Zoster patients are infectious whilst blistering and might cause varicella if in direct contact with a susceptible contact. Thus, zoster could lead to a short-term increased rate of varicella, which would mean that events (i.e. zoster) is more likely to occur in the immediate pre-exposure period. This may artificially inflate the baseline incidence and hence underestimate the relative risk. A valid approach to overcome this scenario is to remove a predefined period of time before exposure from all other unexposed (baseline) time.[8] This has been demonstrated to be valid when exposures arise in a non-homogenous Poisson process and the chance of two or more events occurring in the duration of the pre-exposure period is low.[6]
3. **The event of interest must not censor the observation period**—for example, if the zoster event increases the likelihood of death. This assumption is fulfilled, as zoster is very rarely associated with increased mortality.[9]

Box e-2: **Criteria used to ascertain immunosuppressive conditions and treatments**

The methods used to identify immunosuppressive conditions and therapies that are contraindications of zoster vaccination uptake were adapted from our earlier study of risk factors of zoster.[10] Immunosuppressive conditions included: leukaemia, lymphoma, myeloma, other plasma cell dyscrasias, stem cell and bone marrow transplants, solid organ transplants, Human Immunodeficiency Virus infection and cellular immune deficiency. The immunosuppressive treatments included were: methotrexate, azathioprine, 6-mercaptopurine, biological therapies, steroids, other immunosuppressive agents (e.g. tacrolimus), other disease modifying anti-rheumatic drugs (e.g. ciclosporin), chemotherapy and radiotherapy. We used data on the number of tablets prescribed and the numeric daily dose to calculate the duration of the prescription. As with our previous study, we imputed missing numeric daily dose data, using a "hot-desk" style imputation method. The Table below summarises the immunosuppressive dose criteria and the duration of immunosuppression used for each condition/treatment.

**Immunosuppressive condition or therapy**	**Dose criteria if applicable**	**Period of immune-suppression prior to first medical record**	**Period of immunosuppression after each record**
Lymphoma, myeloma, other plasma cell dyscrasias, leukaemia, bone marrow transplant stem cell transplant	N/A	-	24 months
Cellular immune deficiency, solid organ transplants and HIV	N/A	-	For life
Azathioprine	Immunosuppressed at a dose of ≥50mg/day	3 months[1]	3 months
Methotrexate	Immune-suppressed at dose of>25mg per week (>3.57 mg/day)	3 months[1]	3 months
6-mercaptopurine	Immunosuppressed at a dose of ≥45mg/day	3 months[1]	3 months
Other immunosuppressive agents	Immunosuppressed at any dose	3 months[1]	3 months
Biological agents (e.g. Anti-TNF therapy)	Immunosuppressed at any dose	3 months[1]	12 months
Other disease-modifying anti-rheumatic drugs e.g. mycophenolate, leflunomide	Immunosuppressed at any dose	3 months[1]	3 months
Injectable or oral steroids	Immunosuppressed at >40 mg/day for >7 days, or at >20mg/ day for >14 days	3 months[1]	3 months
Cancer chemotherapy or radiotherapy	Immunosuppressed at any dose	3 months[1]	12 months

[1]3-month period before the first primary care prescription added as the therapy is typically initiated in hospital

Figure e-1: Flow diagram of study participants

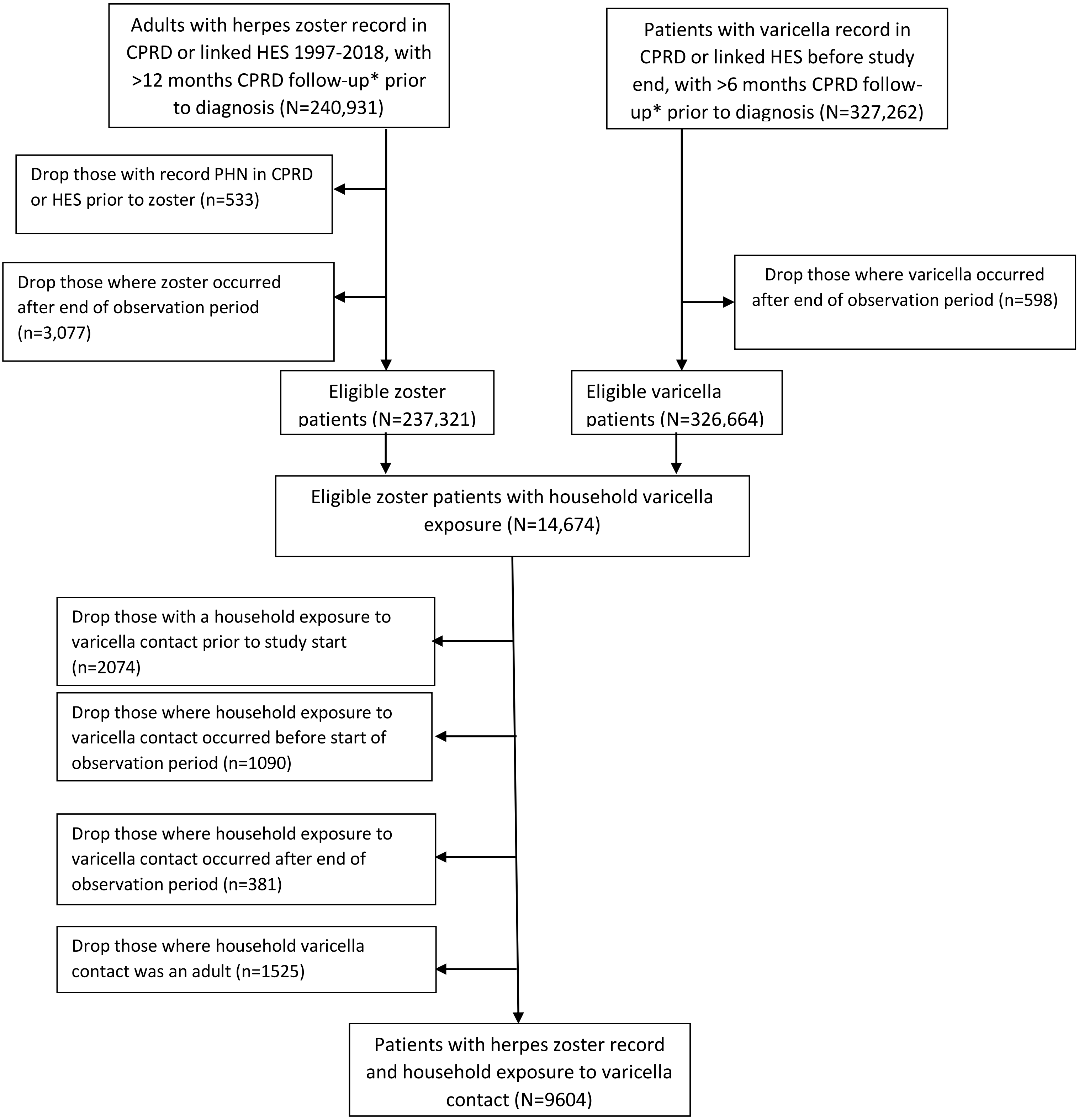

*CPRD follow-up starting at the latest of i) patient registration date at the practice and ii) date when the practice data met CPRD quality control standards[11])

Figure e-2: Distribution of the observation periods for the study participants

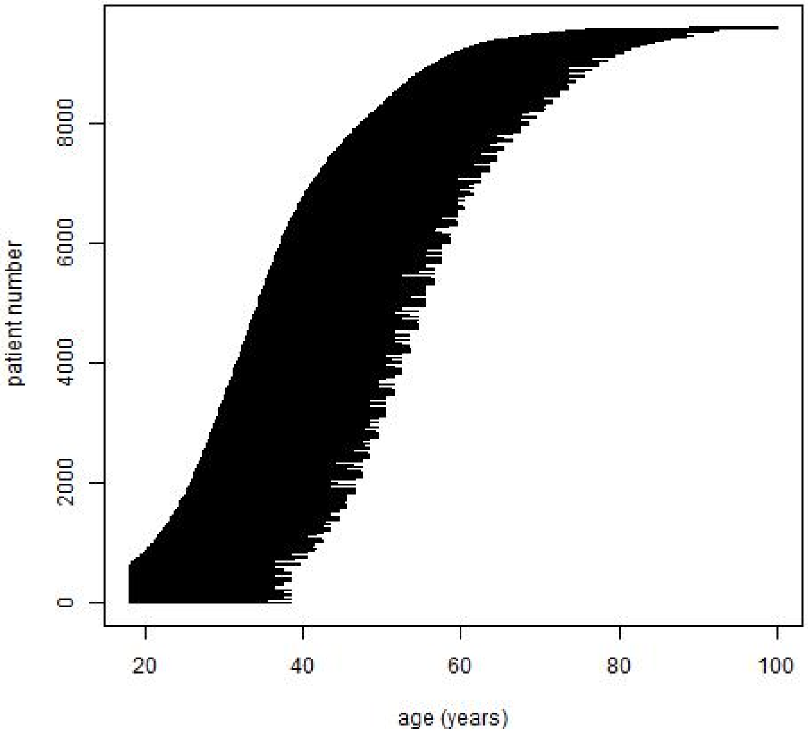

Note: the median (and IQR) length in years of each time-period was: baseline (pre-risk period) - 5.0 (2.3-8.8); risk period - 7.7 (3.8-12.0); and baseline (post-risk period) – 0.5 (0.2-1.0).

Figure e-3: Scatter plot of age at varicella exposure and age at zoster outcome. Vertical dashed lines denote the age group boundaries (when age defined by 40 quantiles of age at event (herpes zoster)).

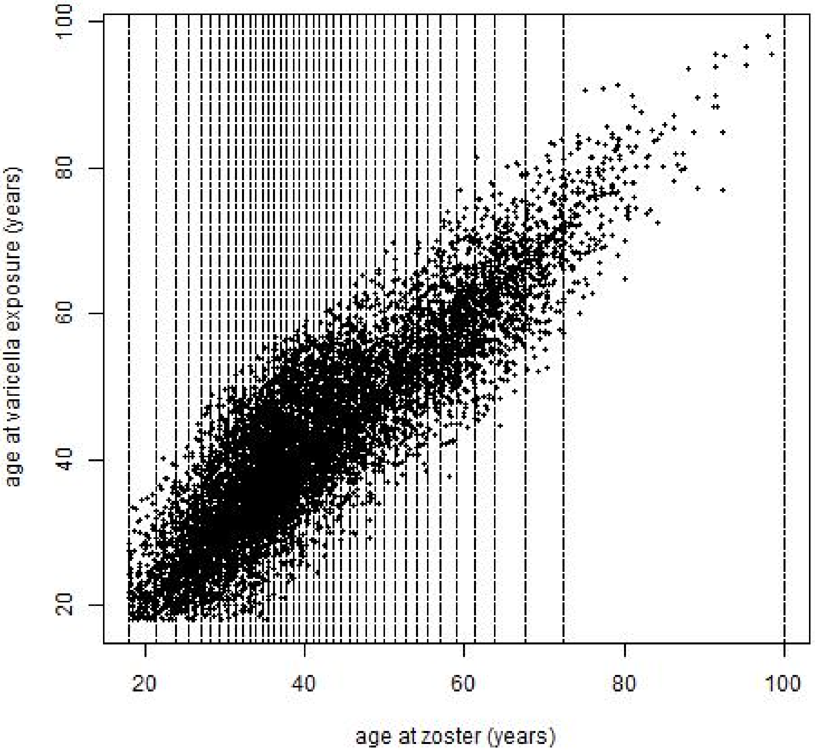

Figure e-4: Graphical representation of the SCCS set-up for an individual: handling multiple exposures

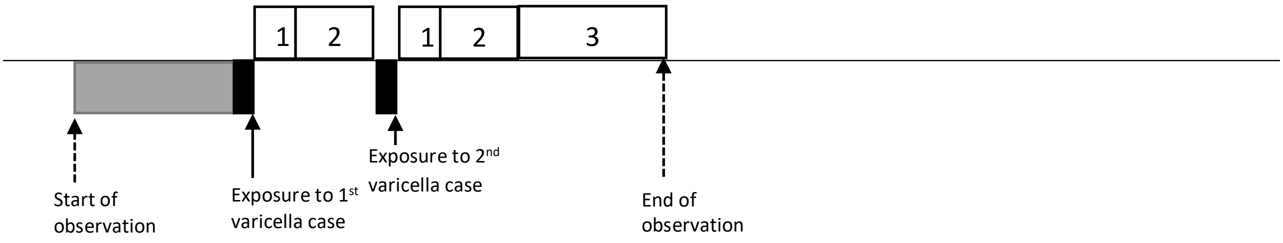

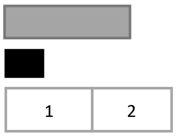

Baseline (unexposed) period

Pre-exposure period 60 days pre-exposure to varicella case

Risk (exposed) period, divided into four risk windows following exposure to varicella case: 1=0-1 years post-exposure; 2=2-4 years post-exposure; 3=5-9 years post-exposure; 4=10-20 years post-exposure.

Table e-1: Characteristics of zoster cases with household gastroenteritis exposure

Characteristic	All zoster cases with household gastroenteritis exposure (N=6577)
Age at outcome, y, median (IQR)	39.2 (32.3-47.7)
Age at first exposure to household gastroenteritis contact, y, median (IQR)	37.4 (31.8-46.1)
Age of household contact at diagnosis of gastroenteritis, y, median (IQR)	2.2 (1.1- 6.4)
Total observation, y, median (IQR)[b]	11.4 (8.5-14.2)
Female sex, N (%)	5494 (71.1)

y: years.

Table e-2: Adjusted incidence ratios for zoster in risk periods following gastroenteritis exposure

Exposure and Risk Period	No. of Cases	Crude IR (95% CI)	Age[2] adjusted IR (95% CI)	Age[2] and calendar[3] period adjusted IR[3] (95% CI)
Acute gastroenteritis				
**Risk period related**				
Baseline[1]	2,655	1.00	1.00	1.00
60 d pre-exposure	123	1.06 (0.88-1.27)	1.01 (0.84-1.21)	1.01 (0.84-1.22)
Risk period				
0-1 y post-exposure	1,274	1.02 (0.95-1.10)	0.95 (0.87-1.04)	0.95 (0.87-1.04)
2-4 y post-exposure	1,236	1.02 (0.94-1.10)	0.90 (0.81-1.01)	0.91 (0.81-1.01)
5-9 y post-exposure	1,019	1.09 (0.99-1.19)	0.88 (0.76-1.04)	0.90 (0.76-1.05)
10-20 y post-exposure	270	1.35 (1.16-1.58)	0.97 (0.76-1.24)	1.00 (0.77-1.28)

Abbreviations: CI, confidence interval; IR, incidence ratio; d: days; y: years.
[1]All time from observation start to 60 days pre-exposure
[2]Age defined by 40 quantiles of age at event (herpes zoster).
[3]Calendar period defined as: 1997-1998, 1999-2000, 2001-2002, 2003-2004, 2005-06, 2007-08, 2009-2010, 2011-2012, as the rotavirus vaccine was introduced in 2013.

Table e-3: Adjusted incidence ratios for zoster in risk periods following varicella exposure, using an indefinite risk period

Exposure and Risk Period	No. of Cases	Crude IR (95% CI)	Age[2] adjusted IR (95% CI)	Age[2], calendar[3] and season[4] adjusted IR (95% CI)
Varicella				
**Risk period**				
Baseline[1]	4109	1.00	1.00	1.00
60 d pre-exposure	433	3.17 (2.87-3.51)	2.90 (2.61-3.22)	2.88 (2.59-3.20)
Risk period				
0-1 y post-exposure	1177	0.77 (0.72-0.83)	0.68 (0.63-0.74)	0.67 (0.62-0.73)
2-4 y post-exposure	1432	0.85 (0.80-0.91)	0.71 (0.65-0.77)	0.69 (0.63-0.76)
5-9 y post-exposure	1546	0.96 (0.89-1.03)	0.71 (0.63-0.80)	0.69 (0.61-0.78)
10-20 y post-exposure	900	1.26 (1.15-1.38)	0.78 (0.65-0.92)	0.74 (0.62-0.88)
>20 years post-exposure	7	2.60 (1.20-5.68)	1.27 (0.57-2.83)	1.21 (0.54-2.74)

Abbreviations: CI, confidence interval; IR, incidence ratio; d: days; y: years.
[1]All time from observation start to 60 days pre-exposure
[2]Age defined by 40 quantiles of age at event (herpes zoster).
[3]Calendar period defined as; 1997-1998, 1999-2000, 2001-2002, 2003-2004, 2005-06, 2007-08, 2009-2010, 2011-2012, 2013-2014, 2015-2016, 2017-2018.
[4]Season defined as; Winter (December to February), Spring (March to May), Summer (June to August) and Autumn (September to November).

Table e-4: Main analysis and sensitivity analyses: Age[1], calendar year and sex-adjusted IR (95% CIs) for zoster in risk periods following varicella exposure (primary analysis), varying some key assumptions

Exposure and Risk Period	MAIN ANALYSIS (N=9,604)	Spline based age-adjusted[3]	Restricted to: patients with linked HES data (N=5,481)	Restricted to: households within a single child under 16 years[4] (N=1,512)	Restricted to: those with only one zoster episode[5] during the observation period (N=9,472)	Restricted to: those never receiving the zoster vaccine[6] (N=9,392)
Varicella						
Baseline[2]	**1.00**	1.00	1.00	1.00	1.00	1.00
60 d pre-exposure	**2.87 (2.58-3.19)**	2.79 (0.51-3.10)	3.08 (2.69-3.53)	3.55 (2.78-4.54)	2.86 (2.58-3.19)	2.89 (2.60-3.21)
20 year risk period						
0-1 y post-exposure	**0.67 (0.62-0.73)**	0.67 (0.62-0.73)	0.70 (0.63-0.78)	0.74 (0.61-0.89)	0.67 (0.62-0.72)	0.67 (0.62-0.73)
2-4 y post-exposure	**0.69 (0.63-0.76)**	0.69 (0.63-0.76)	0.74 (0.66-0.84)	0.66 (0.52-0.83)	0.69 (0.63-0.76)	0.70 (0.64-0.76)
5-9 y post-exposure	**0.69 (0.61-0.77)**	0.69 (0.61-0.77)	0.74 (0.64-0.87)	0.72 (0.53-0.96)	0.69 (0.61-0.77)	0.70 (0.62-0.79)
10-20 y post-exposure	**0.73 (0.62-0.87)**	0.75 (0.63-0.89)	0.83 (0.66-1.05)	0.85 (0.55-1.31)	0.74 (0.63-0.88)	0.75 (0.63-0.89)

Abbreviations: CI, confidence interval; IR, incidence ratio; d: days; y: years.
[1]Age defined by 40 quantiles of age at event (herpes zoster).
[2]All time from observation start to 60 days pre-exposure.
[3]Age effect represented by a linear combination of cubic M-spline function: The smoothing parameter for the age-related relative incidence function was chosen by an approximate cross validation method and the number of interior knots used to define the M-spline basis functions (which are related to the age specific relative incidence function), was 12 knots (the default for the SCCS package).
[4]Defined as households with a single child under 16 years before July 2018.
[5]Second episode defined as zoster record five years after first zoster record).
[6]Zoster vaccination records were determined from data in the immunisation file, Read codes in clinical, test, immunisation and referral files and product codes in the therapy file, as in previous studies.

Table e-5: Characteristics of hip fracture* cases with household varicella exposure

Characteristic	All hip fracture cases with household varicella exposure (N=1,447)
Age at outcome, y, median (IQR)	44.9 (35.8-59.2)
Age at first exposure to household varicella contact, y, median (IQR)	40.5 (33.2-56.6)
Total observation, y, median (IQR)[b]	14.4 (10.6-17.4)
Female sex, N (%)	892 (52.3)

*Hip fracture identified in CPRD or HES and defined as any hip fracture excluding surgical, allograft, autograft, neoplasm-related, or stress-related hip fractures
y: years.

Table e-6: Adjusted incidence ratios for hip fracture* in risk periods following varicella exposure

Exposure and Risk Period	No. of Cases	Crude IR (95% CI)	Age[2] adjusted IR (95% CI)	Age[2] and calendar[3] period adjusted IR[3] (95% CI)	Spline based age-adjusted[4]
Varicella exposure					
**Risk period**					
Baseline[1]	430	1.00	1.00	1.00	1.00
60 d pre-exposure	19	1.44 (0.91-2.29)	1.13 (0.70-1.80)	1.09 (0.68-1.75)	1.05 (0.66-1.68)
20 year risk period					
0-1 y post-exposure	226	1.62 (1.36-1.92)	1.20 (0.98-1.47)	1.16 (0.94-1.42)	1.15 (0.94-1.40)
2-4 y post-exposure	271	1.99 (1.67-2.36)	1.24 (0.98-1.59)	1.21 (0.95-1.55)	1.11 (0.87-1.41)
5-9 y post-exposure	324	3.00 (2.50-3.60)	1.45 (1.05-2.00)	1.42 (1.02-1.98)	1.24 (0.90-1.71)
10-20 y post-exposure	177	4.66 (3.67-5.92)	1.52 (0.96-2.40)	1.56 (0.97-2.52)	1.21 (0.76-1.92)

Abbreviations: CI, confidence interval; IR, incidence ratio; d: days; y: years.
*Hip fracture identified in CPRD or HES and defined as any hip fracture excluding surgical, allograft, autograft, neoplasm-related, or stress-related hip fractures
[1]All time from observation start to 60 days pre-exposure
[2]Age defined by 40 quantiles of age at event (herpes zoster).
[3]Calendar period defined as: 1997-1998, 1999-2000, 2001-2002, 2003-2004, 2005-2006, 2007-2008, 2009-2010, 2011-2012, 2013-2014, 2015-2016, 2017-2018
[4]Age effect represented by a linear combination of cubic M-spline function: The smoothing parameter for the age-related relative incidence function was chosen by an approximate cross validation method and the number of interior knots used to define the M-spline basis functions (which are related to the age specific relative incidence function), was 12 knots (the default for the SCCS package).

Table e-7: Adjusted incidence ratios (IR) for zoster in risk periods following varicella exposure (N=9604), using different time-periods for the pre-exposure period.

Exposure and Risk Period	No. of zoster events	Crude IR (95% CI)	Age[2] adjusted IR (95% CI)	Age[2], calendar[3] and season[4] adjusted IR (95% CI)
Baseline[1]	4209			
**30 d pre-exposure**	340	4.99 (4.46-5.59)	4.55 (4.05-5.11)	4.51 (4.02-5.07)
Risk period				
0-1 y post-exposure	1177	0.76 (0.71-0.82)	0.67 (0.62-0.73)	0.67 (0.62-0.72)
2-4 y post-exposure	1432	0.84 (0.79-0.90)	0.69 (0.64-0.76)	0.68 (0.63-0.75)
5-9 y post-exposure	1546	0.95 (0.88-1.02)	0.69 (0.62-0.78)	0.68 (0.60-0.76)
10-20 y post-exposure	900	1.24 (1.13-1.36)	0.75 (0.64-0.89)	0.72 (0.61-0.85)
Baseline[1]	4042			
**90 d pre-exposure**	507	2.48 (2.25-2.73)	2.25 (2.04-2.49)	2.24 (2.03-2.48)
Risk period				
0-1 y post-exposure	1177	0.77 (0.72-0.83)	0.68 (0.63-0.73)	0.67 (0.62-0.73)
2-4 y post-exposure	1432	0.85 (0.80-0.91)	0.70 (0.64-0.77)	0.69 (0.63-0.75)
5-9 y post-exposure	1546	0.96 (0.89-1.03)	0.70 (0.62-0.79)	0.68 (0.61-0.77)
10-20 y post-exposure	900	1.26 (1.15-1.38)	0.76 (0.65-0.90)	0.73 (0.62-0.86)

Abbreviations: CI, confidence interval; IR, incidence ratio; d: days; y: years.
[1]All time from observation start to 60 days pre-exposure, and 20 years after exposure
[2]Age defined by 40 quantiles of age at event (herpes zoster).
[3]Calendar period defined as; 1997-1998, 1999-2000, 2001-2002, 2003-2004, 2005-06, 2007-08, 2009-2010, 2011-2012, 2013-2014, 2015-2016, 2017-2018.
[4]Season defined as; Winter (December to February), Spring (March to May), Summer (June to August) and Autumn (September to November).

References

1. Yawn BP, Wollan PC, Kurland MJ, et al. Herpes zoster recurrences more frequent than previously reported. *Mayo Clinic proceedings* 2011;86(2):88-93. doi: 10.4065/mcp.2010.0618 [published Online First: 01/10]
2. Hope-Simpson RE. THE NATURE OF HERPES ZOSTER: A LONG-TERM STUDY AND A NEW HYPOTHESIS. *Proceedings of the Royal Society of Medicine* 1965;58:9-20. [published Online First: 1965/01/01]
3. Donahue JG, Choo PW, Manson JE, et al. The incidence of herpes zoster. *Archives of internal medicine* 1995;155(15):1605-9. [published Online First: 1995/08/07]
4. Dworkin RH, Johnson RW, Breuer J, et al. Recommendations for the management of herpes zoster. *Clin Infect Dis* 2007;44 Suppl 1:S1-26. doi: 10.1086/510206 [published Online First: 2006/12/05]
5. Kawai K, Gebremeskel BG, Acosta CJ. Systematic review of incidence and complications of herpes zoster: towards a global perspective. *BMJ Open* 2014;4(6):e004833. doi: 10.1136/bmjopen-2014-004833
6. Whitaker HJ, Farrington CP, Spiessens B, et al. Tutorial in biostatistics: the self-controlled case series method. *Stat Med* 2006;25(10):1768-97. doi: 10.1002/sim.2302
7. Farrington CP, Hocine MN. Within-individual dependence in self-controlled case series models for recurrent events. *Journal of the Royal Statistical Society: Series C (Applied Statistics)* 2010;59(3):457-75. doi: 10.1111/j.1467-9876.2009.00703.x
8. Pratt NL, Roughead EE, Ramsay E, et al. Risk of hospitalization for stroke associated with antipsychotic use in the elderly: a self-controlled case series. *Drugs & aging* 2010;27(11):885-93. doi: 10.2165/11584490-000000000-00000 [published Online First: 2010/10/23]
9. Bricout H, Haugh M, Olatunde O, et al. Herpes zoster-associated mortality in Europe: a systematic review. *BMC public health* 2015;15:466-66. doi: 10.1186/s12889-015-1753-y
10. Walker JL, Andrews NJ, Amirthalingam G, et al. Effectiveness of herpes zoster vaccination in an older United Kingdom population. *Vaccine* 2018;36(17):2371-77. doi: 10.1016/j.vaccine.2018.02.021
11. Herrett E, Gallagher AM, Bhaskaran K, et al. Data Resource Profile: Clinical Practice Research Datalink (CPRD). *International journal of epidemiology* 2015;44(3):827-36. doi: 10.1093/ije/dyv098 [published Online First: 06/06]
